# Subtomogram averages of mitochondrial ATP synthase dimers from plants show a conserved extra density at the peripheral stalk

**DOI:** 10.1107/S2052252525006220

**Published:** 2025-08-21

**Authors:** Thorsten B. Blum, Karen M. Davies, Werner Kühlbrandt

**Affiliations:** a Department of Structural Biology Max Planck Institute of Biophysics Max von Laue Strasse 3 60438Frankfurt am Main Germany; Max Planck Institute of Molecular Physiology, Germany

**Keywords:** ATP synthases, cryo-electron tomography, subtomogram averaging, mitochondria, 3D reconstruction and image processing, structure determination, cryo-electron microscopy, macromolecular machines

## Abstract

We analyzed ATP synthase dimers in mitochondria purified from four plant species by subtomogram averaging, using *Saccharomyces cerevisiae* as a control. The purified mitochondria were plunge-frozen and broken mitochondria were examined by electron cryo-tomography. In each case, the ATP synthase dimers formed extensive rows along the tightly curved cristae ridges. ATP synthase dimer particles of each species were picked and averaged. Subtomogram averages of all four plant ATP synthases revealed an extra density at the tip of the peripheral stalk next to the OSCP hinge that is not seen in *S. cerevisae.* The dimer angle between the ATP synthase monomers is wider than in *Polytomella*, *S. cerevisae* or mammals, but less wide than in *Caenorhabditis elegans*. Slight variations in dimer angle indicate that ATP synthase dimers of plant mitochondria are more flexible than others.

## Abstract 2

Electron cryo-tomography of ATP synthase dimers in plant mitochondria indicates a wide dimer angle and a plant-specific subunit in the peripheral stalk.

## 1. Introduction

Mitochondria are organelles that are found in virtually all eukaryotic cells. They are surrounded by the outer mitochondrial membrane (OMM) and the inner mitochondrial membrane (IMM), separated by the inter-membrane space. Mitochondria are thought to have evolved from free-living bacteria through symbiosis with eukaryotic hosts (Margulis, 1996 ▸; Gray, 2012 ▸). Mitochondria are often called the powerhouses of the eukaryotic cell because the large membrane-protein complexes in the IMM carry out oxidative phosphorylation and produce more than 60% of the cellular ATP (Kim, 2014 ▸).

The mitochondrial respiratory chain consists of membrane-protein complexes I (NADH dehydrogenase), II (succinate dehydrogenase), III (cytochrome *c* reductase) and IV (cytochrome *c* oxidase) (Davies & Daum, 2013 ▸). Electrons are transferred in a series of redox reactions. Electron transfer is coupled to the translocation of protons from the matrix into the cristae space. The resulting proton gradient drives ATP production in the mitochondrial ATP synthase, an F-type ATPase. F-type ATPases are composed of the water-soluble F_1_ subcomplex and the hydrophobic F_o_ motor complex in the membrane. The F_1_ subcomplexes of mitochondrial ATP synthases were first visualized by electron microscopy in 1962 (Fernández-Morán, 1962 ▸) as 9 nm particles on fragments of mitochondrial membranes. Mitochondrial F-ATPases form dimers, which in most cases induce strong membrane curvature (Kühlbrandt, 2019 ▸) and associate into extensive rows (Blum *et al.*, 2019 ▸). In some microorganisms, the mitochondrial ATPase associates into higher-order assemblies. In *Tetrahymena*, the mitochondrial ATP synthase associates into tetramers that give the cristae the characteristic tubular morphology found in these ciliates (Flygaard *et al.*, 2020 ▸). In apicomplexans, such as *Toxoplasma gondii* or the malaria-causing *Plasmodium* spp., tubular cristae are capped by pentagonal pyramids of mitochondrial ATP synthase that induce high local membrane curvature, contributing to the unique bulbous cristae morphology of these pathogenic parasites (Mühleip *et al.*, 2021 ▸). In *Euglena gracilis* it has been shown that the mitochondrial ATP synthase dimers are tightly associated with cardiolipin (Mühleip *et al.*, 2019 ▸). The dimer angle between the long axes of monomers within a dimer varies depending on the species. Dimer angles ranging from 0° in ciliates (Mühleip *et al.*, 2016 ▸) to 105° in the worm *Caeno­rhabditis elegans* (Buzzard *et al.*, 2024 ▸) have been reported.

An initial study of F-ATPase dimers in the IMM of plant mitochondria indicated major differences compared with the structures of other known F-type ATPases (Davies *et al.*, 2011 ▸). For the present work, we purified mitochondria from four different plant species and investigated them by electron cryo-tomography (cryo-ET). Structures of dimers from each species were obtained by subtomogram averaging. Our results demonstrate that ATP synthase dimers from different plant species resemble each other closely, as expected. They form long rows in the IMM, as do all mitochondrial ATP synthases. However, the dimer angle is considerably larger than in most other F-ATPase dimers, and the monomers have an extra density at the peripheral stalk, indicating a different subunit composition.

## 2. Methods

### 2.1. *Arabidopsis* tissue culture

Pieces of a callus culture of *Arabidopsis thaliana* (L.) Heynh. (PC-0011, Deutsche Sammlung von Mikroorganismen und Zellkulturen) were added to a small volume of ZM medium (1650 mg l^−1^ NH_4_NO_3_, 1900 mg l^−1^ KNO_3_, 180.54 mg l^−1^ MgSO_4_, 170 mg l^−1^ KH_2_PO_4_, 332.02 mg l^−1^CaCl_2_, 36.7 mg l^−1^ FeNaEDTA, 6.2 mg l^−1^ H_3_BO_3_, 16.9 mg l^−1^ MnSO_4_·H_2_O, 8.6 mg l^−1^ ZnSO_4_·7H_2_O, 0.83 mg l^−1^ KI, 0.25 mg l^−1^ Na_2_MoO_4_·2H_2_O, 0.025 mg l^−1^ CuSO_4_·5H_2_O, 0.025 mg l^−1^ CoCl_2_·6H_2_O, 1 mg l^−1^ nicotinic acid, 10 mg l^−1^ thiamine hydrochloride, 1 mg l^−1^ pyridoxal hydrochloride, 100 mg l^−1^*myo*-inositol, 1 mg l^−1^ 2,4-dichlorophenoxyacetic acid, 30 g l^−1^ sucrose) and shaken at 25°C at 100 rev min^−1^ for several weeks to obtain a homogenous suspension culture. At this stage, agitation was stopped after one week. The cells were left to sediment for a few minutes and the volume of the suspension with a high cell content was measured. Fresh ZM medium was added to a total volume of medium equal to the measured volume and the cells were shaken again. In the second week the cells were sedimented again and the old medium was removed. Fresh ZM medium equal to the measured volume was added and the cells were resuspended. This procedure was repeated every two weeks until the culture was harvested.

### 2.2. Isolation of plant mitochondria

For an overview of the isolation procedures, see Supplementary Fig. S1. Onions (*Allium cepa*) and white asparagus (*Asparagus officinalis*) were purchased at a local supermarket. Onion tubers were peeled and cut. Asparagus shoots were washed and cut into short segments. Sunflower seeds (*Helianthus annuus*) were sterilized for 10 min in 3%(*v*/*v*) NaClO before an overnight wash in water. The following day, the seeds were placed between layers of humidified paper towels. The seeds were left to germinate in the dark at 21°C. Etiolated seedlings were harvested after ten days. *A. thaliana* cells from suspension cultures were pelleted (18°C, 3000*g*, 5 min).

All subsequent steps were performed at 4°C. The plant material was mixed with an equal amount (*w*/*v*) of ice-cold grinding buffer [for onion, asparagus and sunflower, 400 m*M* mannitol, 25 m*M* MOPS–KOH pH 7.8, 10 m*M* EDTA, 10 m*M* DTT, 1%(*w*/*v*) PVP-40, 0.01%(*w*/*v*) BSA (fatty acid-free), one Complete Protease Inhibitor Cocktail tablet tablet per litre; for *Arabidopsis*, 300 m*M* sucrose, 25 m*M* MOPS–KOH pH 7.2, 1 m*M* EGTA, 2.5 m*M* DTT, 0.01%(*w*/*v*) BSA (fatty acid-free), one Complete Protease Inhibitor Cocktail tablet per litre]. The material was homogenized in a Waring blender three times for 3 s each. The homogenate was filtered through four layers of muslin. Because the isolation procedure disrupted the acidic cell vacuole, the pH was adjusted to 7.2–7.4 with 10 *M* KOH (Braun *et al.*, 1992 ▸).

Plant mitochondria were isolated as described by Boutry *et al.* (1984 ▸) and Braun *et al.* (1992 ▸) with minor modifications. All steps were performed at 4°C. Broken cells were centrifuged (4°C, 1000*g*, 5 min) to remove starch, cell-wall fragments and unbroken cells. Mitochondria were pelleted from the supernatant (4°C, 12 000*g*, 15 min) and resuspended in washing buffer (for potato, asparagus and sunflower, 400 m*M* mannitol, 10 m*M* MOPS–KOH pH 7.4, 1 m*M* EDTA; for *Arabidopsis*, 300 m*M* sucrose, 10 m*M* MOPS–KOH pH 7.2, 1 m*M* EDTA; 350 ml buffer per 1000 g starting material). The remaining starch, cell-wall fragments and unbroken cells were removed by centrifugation (4°C, 1000*g*, 5 min) and the mitochondria were pelleted from the supernatant by a second centrifugation step (4°C, 12 000*g*, 15 min). The mitochondrial pellet was resuspended in washing buffer (25 ml buffer per 1000 g starting material) and the procedure was repeated with a further low-speed centrifugation step (4°C, 1000*g*, 5 min). The mitochondria were sedimented by high-speed centrifugation (4°C, 12 000*g*, 15 min) and resuspended in a small volume of washing buffer (4 ml buffer per 1000 g starting material).

The mitochondria were further purified with a self-generated Percoll density gradient. 50 ml washing buffer containing 28% Percoll was pipetted into a tube for the Ti45 ultracentrifuge rotor and overlaid with 2–4 ml suspended mitochondria. The Percoll gradient formed during centrifugation at 37 600*g* for 45 min at 4°C. The mitochondria accumulated in a band in the lower half of the gradient. The mitochondria band was extracted and diluted with washing buffer to reduce the Percoll content to below 5%. The mitochondria were pelleted (4°C, 12 000*g*, 12 min), resuspended in 50 ml washing buffer, pelleted again (4°C, 12 000*g*, 12 min) and washed a further two times with 10 ml SEM buffer (250 m*M* sucrose, 10 m*M* MOPS–KOH pH 7.2, 1 m*M* EDTA). Finally, the mitochondria were resuspended in SEM buffer at a protein concentration of 4–5 mg ml^−1^. The suspension was used for the preparation of EM grids.

### 2.3. Isolation of *S. cerevisiae* mitochondria

Glycerol stocks were used to inoculate 15 ml YPAG medium containing 100 µg ml^−1^ carbenicillin. The culture was grown overnight at 30°C with shaking (130 rev min^−1^). 48 h later, 1 ml was used to inoculate 2 l YPAG medium containing 100 µg ml^−1^ carbenicillin. The culture was grown at 30°C with shaking (120 rev min^−1^) for around 96 h until an OD_600_ of 4–10 was reached and the cells were harvested by centrifugation (18°C, 3000*g*, 5 min).

The pellet was washed in water and pelleted (18°C, 3000*g*, 5 min). The cells were washed a second time and pelleted (18°C, 3000*g*, 5 min). The following steps were performed for 1 g of wet pellet. The pellet was resuspended in 2 ml DTT buffer (100 m*M* Tris–H_2_SO_4_ pH 9.4, 10 m*M* DTT) and incubated with gentle agitation at 30°C for 15 min. The cells were harvested by centrifugation (18°C, 1500*g*, 5 min) and washed in 6 ml sorbitol buffer (20 m*M* K_2_HPO_4_–H_3_PO_4_ pH 7.4, 1.2 *M* sorbitol). The cells were pelleted (18°C, 1500*g*, 5 min) and resuspended in 9 ml sorbitol buffer. The OD_600_ was determined from 5 µl cell suspension in 1 ml water. 3 mg of Zymolyase 20T was added to 9 ml of the suspension and incubated with gentle agitation at 30°C. Every 10 min, 5 µl of the suspension was mixed with 1 ml water and the OD_600_ was checked. When the OD_600_ was 30–40% of the starting value (after ∼30 min) the spheroplasts that had formed due to the digestion of the cell wall with Zymolyase were harvested (4°C, 1500*g*, 5 min).

All subsequent steps were carried out at 4°C or on ice. The spheroplasts were resuspended in 6 ml ice-cold buffer *A* [20 m*M* Tris–HCl pH 7.4, 600 m*M* sorbitol, 1 m*M* EDTA, 0.2%(*w*/*v*) BSA (fatty acid-free), one Complete Protease Inhibitor Cocktail Tablet per litre], transferred to a glass homogenizer and broken with 15 strokes of a tight-fitting pestle. 6 ml buffer *A* was added and unbroken cells, nuclei and large debris were removed by centrifugation (4°C, 1000*g*, 5 min). Mitochondria were harvested from the supernatant (4°C, 12 000*g*, 15 min) and resuspended in 2 ml buffer *A*. Unbroken cells, nuclei and cell debris were removed by centrifugation (4°C, 1500*g*, 5 min). The mitochondria were pelleted from the supernatant (4°C, 12 000*g*, 15 min), resuspended in 2 ml buffer *A* and cell debris was removed by a third centrifugation (4°C, 3000*g*, 5 min). The mitochondria in the supernatant were pelleted again (4°C, 12 000*g*, 15 min), resuspended in 20 µl SEM buffer (10 m*M* MOPS–KOH pH 7.2, 250 m*M* sucrose, 1 m*M* EDTA), frozen in liquid nitrogen and stored at −80°C.

The mitochondria were further purified with a sucrose step gradient (Supplementary Fig. S1). EM buffers (10 m*M* MOPS–KOH pH 7.2, 1 m*M* EDTA) containing varying concentrations of sucrose were added to a 14 ml SW40 centrifuge tube starting with the heaviest sucrose solution (1.5 ml EM buffer with 60% sucrose, 4 ml EM buffer with 32% sucrose, 1.5 ml EM buffer with 23% sucrose and 1.5 ml EM buffer with 15% sucrose). 2 ml of the mitochondria suspension was layered on top of the gradient and centrifuged at 4°C at 13 000 g for 60 min. The mitochondria migrated to the 60/32% sucrose interface.

The mitochondrial band at the 60/32% sucrose interface was extracted and diluted with SEM buffer to reduce the sucrose concentration to less than 5%. The mitochondria were pelleted (4°C, 12 000*g*, 12 min), resuspended in 50 ml SEM buffer, pelleted (4°C, 12 000*g*, 12 min) and washed twice more with 10 ml SEM buffer. Finally, the mitochondria were resuspended with SEM buffer to a protein concentration of 4–5 mg ml^−1^. This mitochondria suspension was used for cryo-EM grid preparation.

### 2.4. Electron cryo-tomography and subtomogram averaging

Electron cryo-tomography was performed as described by Davies *et al.* (2014 ▸). 3 µl of mitochondria mixed 1:1 with fiducial markers (10 nm gold particles conjugated to protein A; Aurion) were applied onto glow-discharged Quantifoil grids (R2/2, Cu 300 mesh, Quantifoil), blotted for 3 s to remove excess liquid (#4 Whatman paper) and plunge-frozen in liquid ethane using a home-built freezing device. Single-axis tilt series (±60°) were collected from broken mitochondria containing IMM vesicles on an FEI Krios or a Jeol 3200 FSC transmission electron microscope operating at 300 kV and equipped with a post-column energy filter (GIF Quantum, Gatan in the Krios or an in-column Omega filter in the Jeol). Both microscopes were equipped with a K2 Summit detector (Gatan) operating in counting mode. Tilt series were acquired with the *Latitude* software (Gatan) using a specimen pixel size of 0.33–0.43 nm and a defocus of 2.2–5.5 µm. Tilt series were aligned using the gold fiducial markers and back-projected to generate tomographic volumes using the *IMOD* software (Kremer *et al.*, 1996 ▸). ATP synthase dimers were averaged as described in Davies *et al.* (2012 ▸). *C*2 symmetry was applied, with one monomer of the dimer undergoing refinement and averaging. The resulting averaged monomers were subsequently placed into tomographic volumes using the *AMIRA* EM toolbox (Pruggnaller *et al.*, 2008 ▸). Membranes were manually segmented in *AMIRA* (FEI). The angle between the two monomers within each dimer was determined based on the Euler angles of the refined monomers.

## 3. Results

### 3.1. Mitochondrial plant ATP synthase forms dimer rows in inner membrane cristae

**Figure 1:**
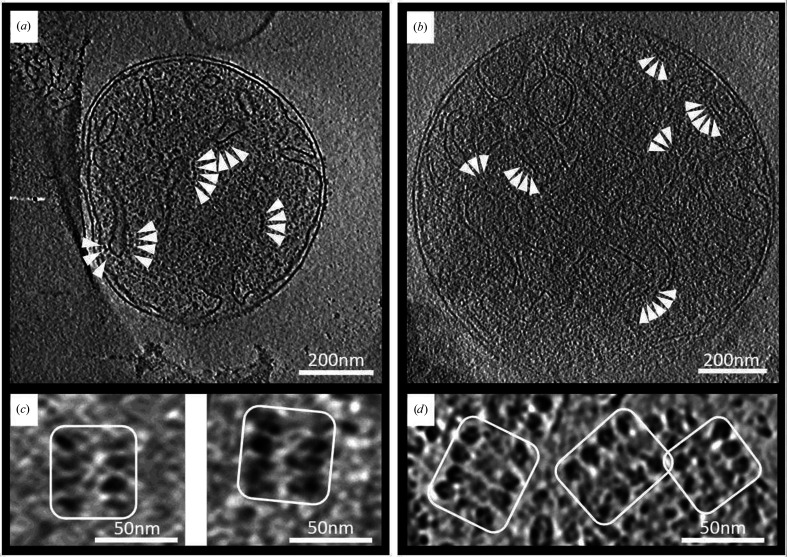
Cryo-ET of isolated mitochondria. Inner membrane cristae are clearly visible in tomographic slices of intact mitochondria from *Arabidopsis* (*a*) and *Asparagus* (*b*) (white arrowheads). Rows of ATP synthase dimers were apparent in slices showing top views of cristae ridges in broken mitochondria of both plant species (*c*, *d*) (white boxes). In broken mitochondria, the contrast is higher due to the absence of the mitochondrial matrix, but the arrangement of protein complexes in the membrane is unchanged.

Mitochondria from the four plant species *Helianthus annuus* (sunflower), *Allium cepa* (onion), *Asparagus officinalis* (asparagus) and *Arabidopsis thaliana* (thale cress) were purified (Supplementary Fig. S1) and investigated by cryo-ET (Supplementary Fig. S2). In parallel, mitochondria from the yeast *Saccharomyces cerevisiae* were examined in the same way as a control. The mitochondria ranged in size between 0.6 and 1.15 µm in the longest dimension. The OMM and IMM were clearly visible as dark lines in slices of tomographic volumes (Fig. 1 ▸). Mitochondria were packed with IMM cristae. In the cristae, rows of ATP synthase dimers were identified. Broken mitochondria that had lost most of their internal matrix were selected to obtain tomographic volumes with high contrast and a good signal-to-noise ratio. Rows of ATP synthase dimers were easily detected in IMM vesicles of broken mitochondria.

### 3.2. Subtomogram average volumes

**Figure 2:**
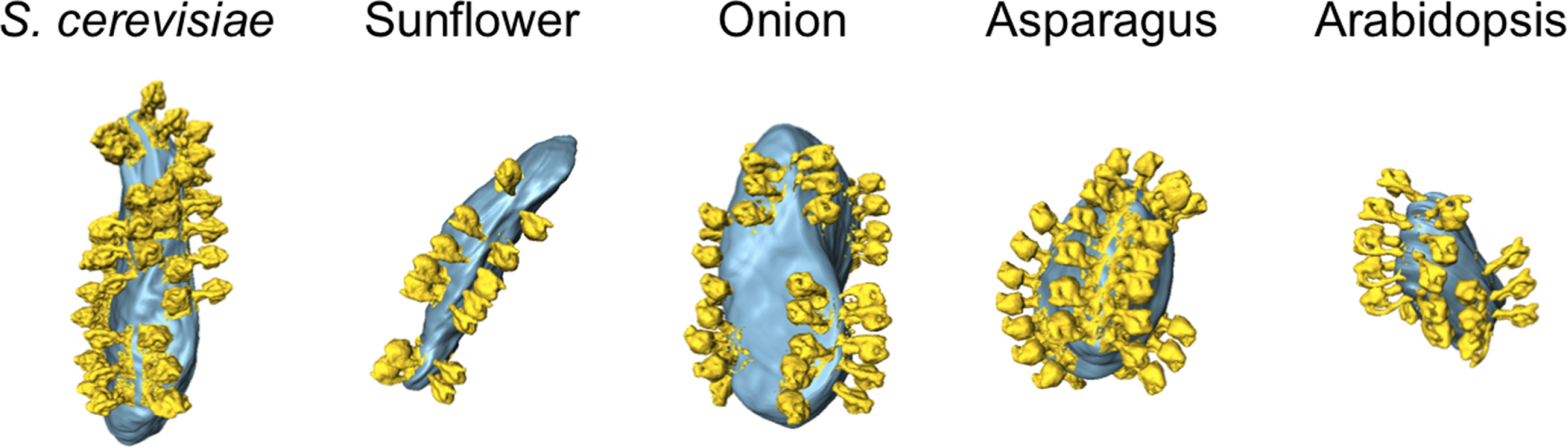
ATP synthase monomer volumes placed back into the tomographic volume. ATP synthase dimers (yellow) form rows on highly curved membrane vesicles (light blue) in each investigated plant species.

In tomograms of each of the four plant species and yeast, between 92 and 504 ATP synthase dimer volumes were picked, aligned and averaged. Subsequently, each monomer of the averaged dimers was independently averaged and placed back into the tomographic volumes, which were later segmented (Fig. 2 ▸). The ATP synthase dimers, consisting of two monomers, form rows in regions of high membrane curvature, with the peripheral stalks of the ATP synthase monomers facing each other.

**Figure 3:**
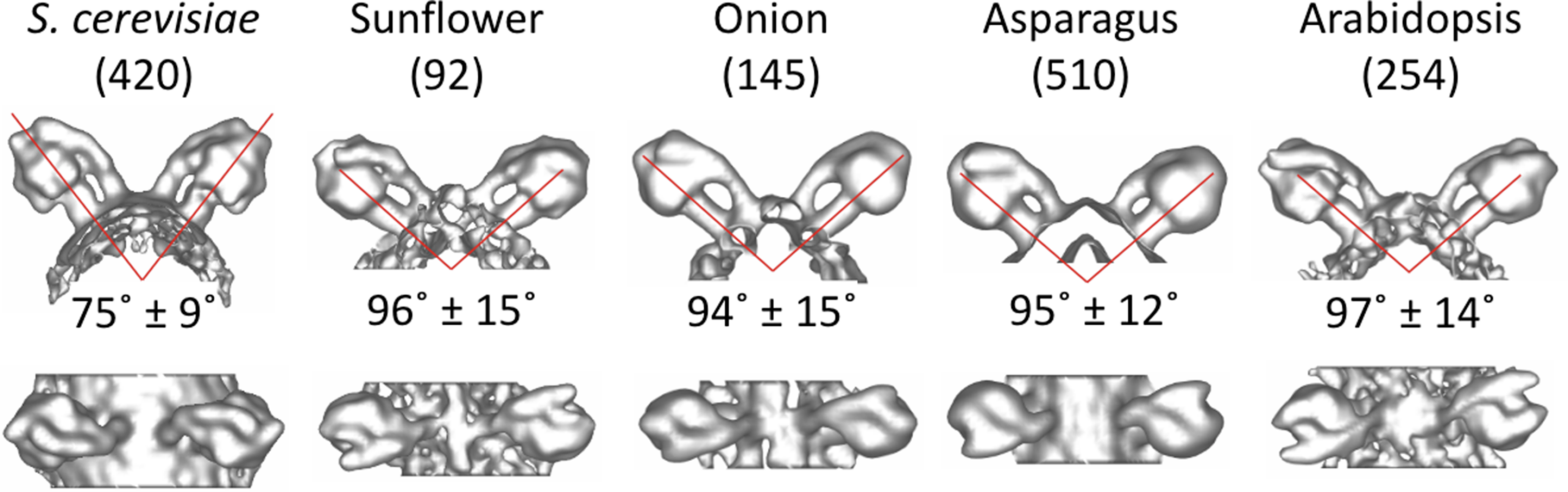
Structures of ATP synthase dimers. The membrane portion of the peripheral stalk in ATP synthase dimers consistently aligns with the membrane segment of the central stalk. The main difference between species is the angle between the ATP synthase monomers. The angle varies between ∼75° for *S. cerevisiae* and ∼96° for plants.

Inevitably, the averaged volumes contained some false positives or mis-picked dimers. These were identified by placing the average volumes back into the tomogram according to the rotation and displacement parameters determined during alignment. Mis-picked dimers then showed up as poorly aligned particles in the otherwise well defined and regular dimer rows. Misaligned particles were excluded during refinement, enhancing the resolution of the final subtomogram averages (Fig. 3 ▸).

### 3.3. Dimer angle

**Figure 4:**
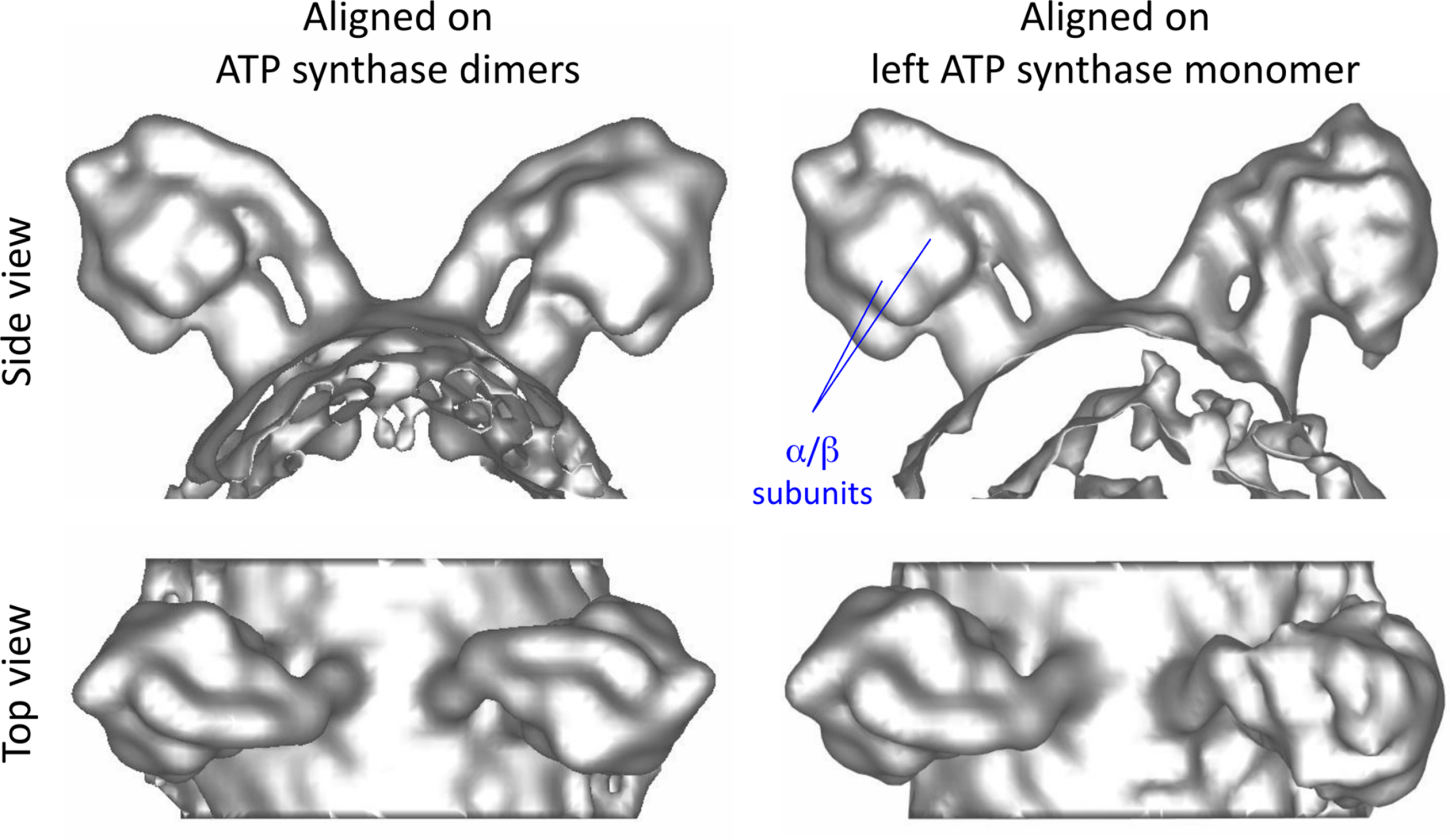
Flexibility of ATP synthase monomers in ATP synthase dimers. The ATP synthase dimer is not rigid, and the angle between two ATP synthase monomers in the dimer varies slightly. When only a single monomer of an aligned ATP synthase dimer is used as a reference (in this case that from *S. cerevisiae*), the resolution of the other monomer decreases and structural features, in particular the distinct α and β subunits of the catalytic F_1_ head, are blurred.

The angle between the long axis of the ATP synthase monomers in dimers of plant mitochondria was determined as ∼96° (Fig. 3 ▸). We found that the dimer angle varies by ±15° (Fig. 3 ▸). This becomes obvious when the volumes are aligned with one ATP synthase monomer in the dimer and then averaged (Fig. 4 ▸). Map features that are clear in the aligned monomer, for example the α and β subunits in the F_1_ head, are lost in the non-aligned monomer.

**Figure 5:**
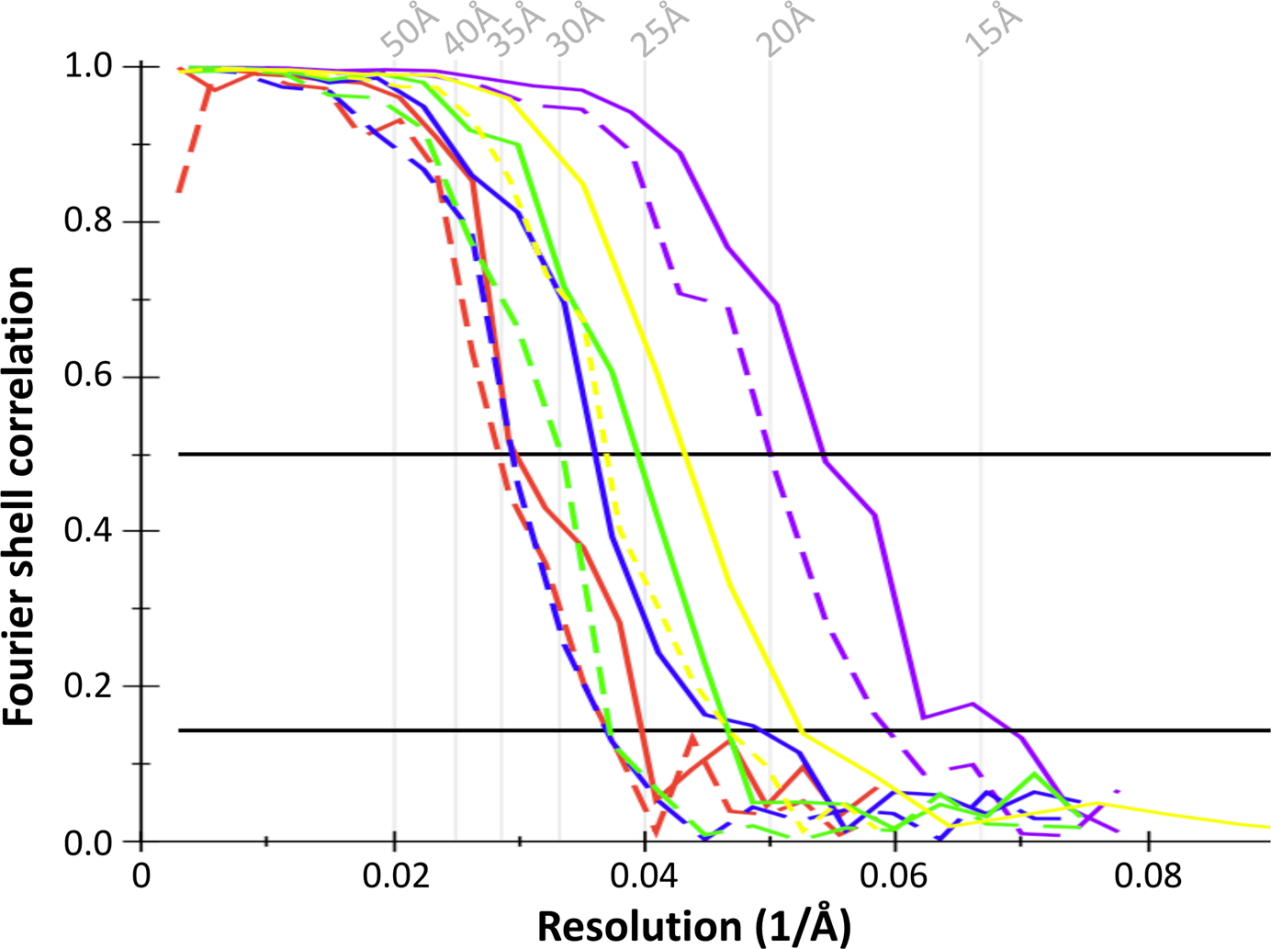
Fourier shell correlations of ATP synthase dimers (dashed lines) and monomers (solid lines). The resolutions of the different maps were estimated using the Fourier shell correlation with the 0.5 criterion. The resolution improves from left (onion) to right (yeast). The resolution of ATP synthase monomer volumes is better than for the dimer volumes because the average is not affected by variations in dimer angle.

The resolutions of the different mitochondrial ATP synthase monomers were calculated using the FSCs and plotted (Fig. 5 ▸). The estimated resolutions of the subtomogram average volumes range from 19 to 31 Å, depending on the pixel size and the number of particles averaged (Table 1 ▸).

### 3.4. ATP synthases from plant mitochondria have an extra peripheral stalk density

**Figure 6:**
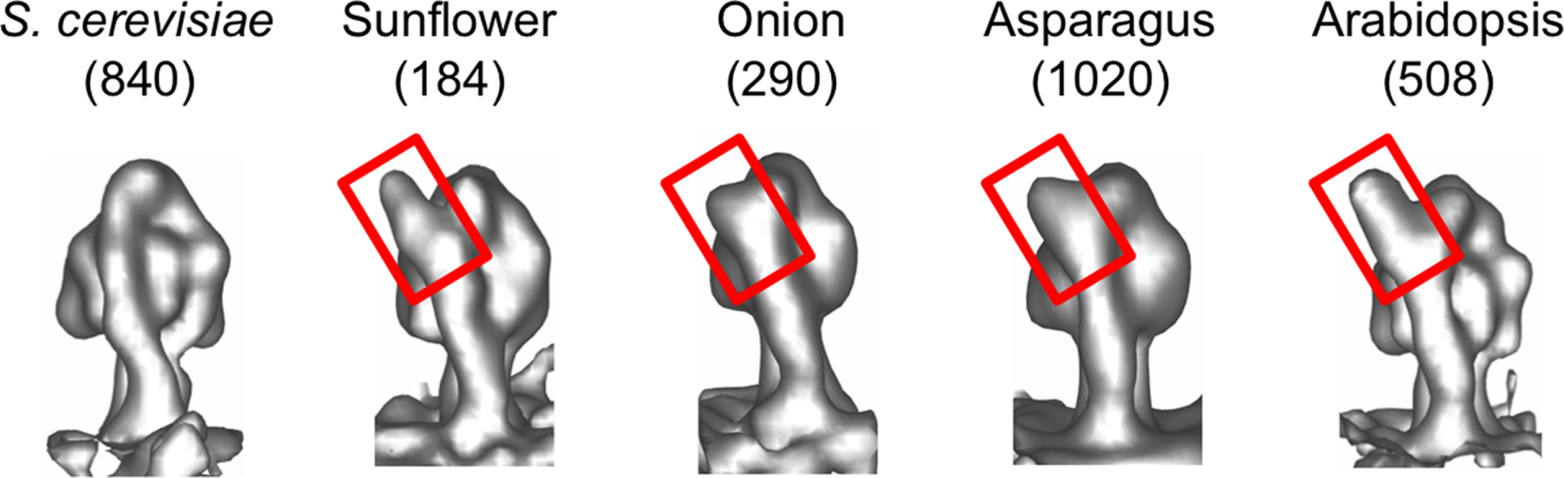
Structures of ATP synthase monomers. Plant ATP synthase monomers exhibit specific structural alteration compared with those from *S. cerevisiae*. The plant ATP synthases have an extra density at the apex of the peripheral stalk (red boxes).

When the dimer volumes are aligned on one ATP synthase monomer, the average volume of this monomer goes to higher resolution, because the map detail is not affected by variations in the dimer angle. In all four ATP synthases from plant mitochondria averaged in this way, an extra density appears at the upper end of the peripheral stalk that is absent in *S. cerevisiae*. The extra density is clearly visible when the monomer maps are viewed from a direction where the peripheral stalk is in front, with the F_1_ head at the back (Fig. 6 ▸). The role and exact structure of this extra subunit or domain is at present unknown.

## 4. Discussion

The ATP synthase dimer angle has a clear and strong effect on dimer row formation, cristae geometry and hence, presumably, on mitochondrial physiology. For example, the minimal dimer angle of 0° in *Paramecium* results in helical arrays and long tubular cristae with small, circular cristae junctions (Mühleip *et al.*, 2016 ▸). In yeasts, the dimer angle of ∼75° results in flat lamellar cristae, with tightly curved edges defined by a single row of dimers and slit-like crista junctions (Davies *et al.*, 2011 ▸; Bohnert *et al.*, 2015 ▸).

In *Polytomella*, the dimer angle of 56° (Blum *et al.*, 2019 ▸) is in between those of ciliates (0°) and yeasts or mammals (∼75°). Correspondingly, *Polytomella* dimers assemble into rows with an intermediate, quasi-helical geometry (Dietrich *et al.*, 2024 ▸). Parallel double rows wrapping around the curved cristae ridges result in club-shaped cristae (Blum *et al.*, 2019 ▸).

The high dimer angle of 105° in *C. elegans* (Buzzard *et al.*, 2024 ▸) is thought to produce particularly narrow cristae, which may be an advantage for efficient use of the shallow pH gradient across the IMM under changing environmental conditions. Similar considerations may apply to plant mitochondria. The mean dimer angle of ∼96° will result in similarly narrow cristae, which may enable plant mitochondria to adapt to pH fluctuations caused by rapid environmental changes, such as variations in ion concentration due to signaling, reproduction or stress (Sze & Chanroj, 2018 ▸; Behera *et al.*, 2018 ▸). In an earlier study (Davies *et al.*, 2011 ▸) we reported an angle of 115° for ATP synthase dimers from potato mitochondria. On reassessing these preliminary data we conclude that this unusually high angle resulted from an uneven, narrow distribution of dimer orientations relative to the direction of the electron beam. In our present analysis, we avoided this source of error by picking dimers with a wider, more even orientation distribution.

The extra density that characterizes plant mitochondrial ATP synthase (Fig. 5 ▸) appears to be adjacent to the OSCP subunit that is found in this position in mitochondrial ATP synthases of other organisms (Murphy *et al.*, 2019 ▸). The OSCP subunit, equivalent to the delta subunit in chloroplast ATP synthase, acts as a flexible hinge between the F_1_ head and the peripheral stalk. Complexome profiling of mitochondrial ATP synthase from the model plant *Arabidopsis* (Röhricht *et al.*, 2021 ▸) identified the plant-specific subunit ATP-F_A_d as a homologue of subunit ASA4 in *Polytomella* (Murphy *et al.*, 2019 ▸). Since ASA4 sits at the tip of the peripheral stalk in the high-resolution single-particle cryo-EM structure of the *Polytomella* ATP synthase dimer (Murphy *et al.*, 2019 ▸), we conclude that the new density adjacent to OSCP in our sub­tomogram averages is the plant-specific subunit ATP-F_A_d. Its location in the peripheral stalk suggests that it might affect the flexibility of the hinge and hence the efficiency or rate of ATP synthesis.

In summary, we conclude that mitochondrial ATP synthase dimers from plants form rows in highly curved cristae membrane regions with a dimer angle of ∼96°. This angle is different from those observed in all other mitochondrial ATP synthase dimers so far, and the dimer angle in ATP synthases from plant mitochondria is more variable than in other clades; ATP synthases from plants also have an extra density on top of the peripheral stalk, which may affect the flexibility of the OSCP hinge.

## Supplementary Material

Supplementary figures. DOI: 10.1107/S2052252525006220/rq5014sup1.pdf

## Figures and Tables

**Table 1:** Resolution comparison of different ATP synthase monomers

Species	Picked monomers	Defocus (µm)	Pixel size (Å)	Resolution (Å)
Onion	290	−5.5	4.277	31
Sunflower	184	−4.0	3.346	29
*Arabidopsis*	508	−2.2	3.346	27
Asparagus	1004	−4.0	4.277	25
Yeast	840	−3.0	3.216	19
