# Supplementary material for: Subtomogram averages of mitochondrial ATP synthase dimers from plants show a conserved extra density at the peripheral stalk
Source: IUCrJ. 2025 Aug 21;12(Pt 5):563–9. doi: 10.1107/S2052252525006220 (PMC12403170; doi:10.1107/S2052252525006220)
Supplement: Supplementary file 1 [file m-12-00563-sup1.pdf]

IUCrJ

Volume 12 (2025)

Supporting information for article:

Subtomogram averages of mitochondrial ATP synthase dimers from plants show a conserved extra density at the peripheral stalk

Thorsten B. Blum, Karen M. Davies and Werner Kühlbrandt

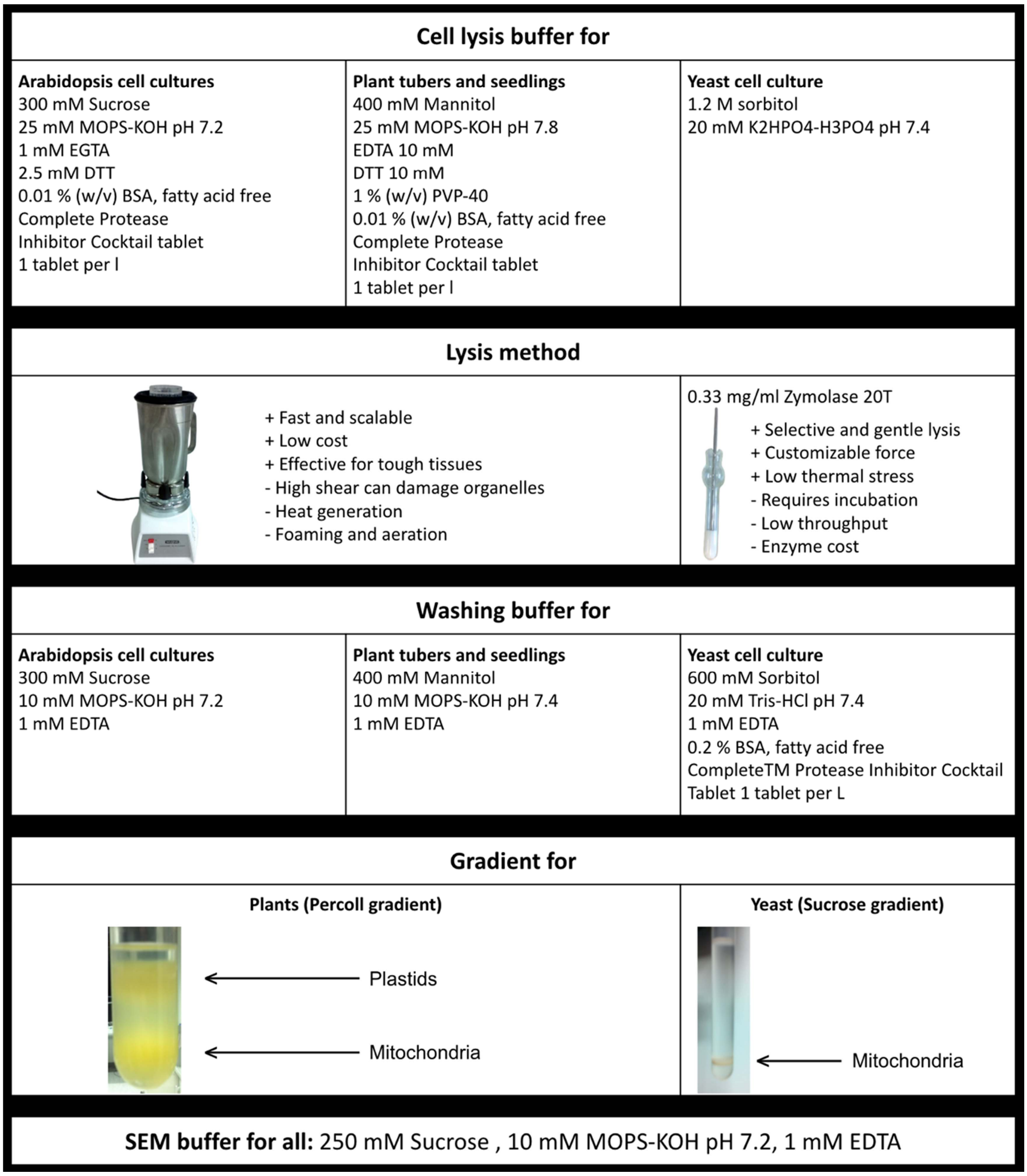

Figure S1 Purification of mitochondria. Overview of buffers and lysis conditions. Lysis buffers were optimized for each species and vary slightly. Note that this procedure normally yields broken mitochondria, unless special precautions are taken, which was not the case. Rupture of the outer membrane does not affect the dimer rows and inner membrane topography, as indicated by comparing cryo-tomograms of ruptured (Blum *et al.*, 2019) and intact *Polytomella* mitochondria (Dietrich *et al.*, 2024). After lysis, mitochondria were washed and further purified by gradient centrifugation. We used Percoll gradients for all plant mitochondria, and sucrose gradients for yeast mitochondria. All samples were ultimately resuspended in the same buffer for cryo-EM specimen preparation.

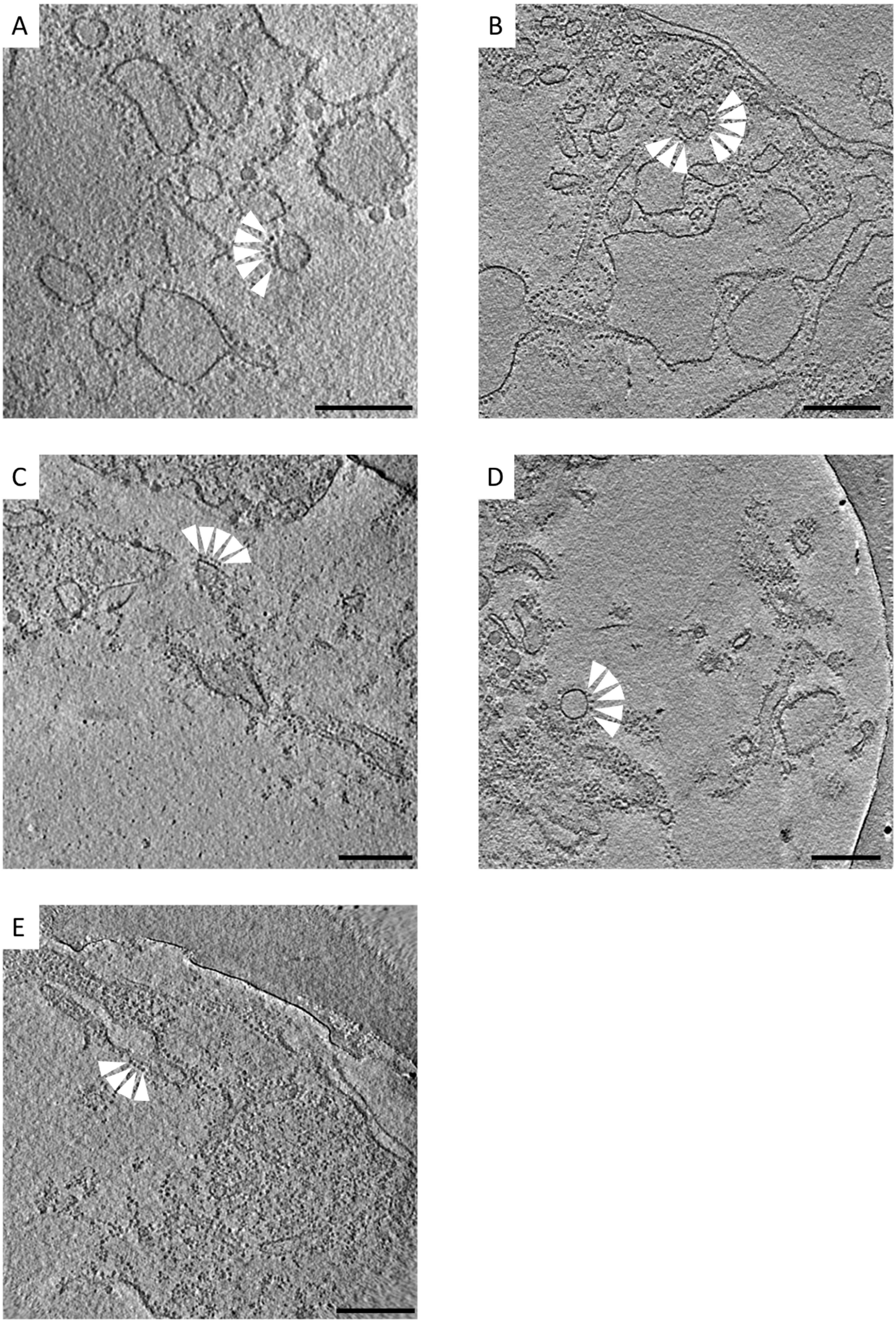

Figure S2 Cryo-electron tomography of plant mitochondria. Representative tomograms of inner mitochondrial membranes isolated from *Arabidopsis* (A), asparagus (B), onion (C), sunflower seedlings (D), and yeast (E). White arrowheads indicate ATP synthase dimers. Scale bars, 200 nm.
